# Nuclear envelope structural proteins facilitate nuclear shape changes accompanying embryonic differentiation and fidelity of gene expression

**DOI:** 10.1186/s12860-017-0125-0

**Published:** 2017-01-14

**Authors:** Elizabeth R. Smith, Yue Meng, Robert Moore, Jeffrey D. Tse, Arn G. Xu, Xiang-Xi Xu

**Affiliations:** Department of Cell Biology, Sylvester Comprehensive Cancer Center, University of Miami Miller School of Medicine, Papanicolaou Building, Room 415 [M877] 1550 NW 10th Avenue, Miami, FL 33136 USA

**Keywords:** Mouse ES cells, Retinoic acid, Lamin A/C, Emerin, Nuclear envelope, Endoderm

## Abstract

**Background:**

Nuclear size and shape are specific to a cell type, function, and location, and can serve as indicators of disease and development. We previously found that lamin A/C and associated nuclear envelope structural proteins were upregulated when murine embryonic stem (ES) cells differentiated to primitive endoderm cells. Here we further investigated the morphological changes of nuclei that accompany this differentiation.

**Results:**

The nuclei of undifferentiated wild type cells were found shaped as flattened, irregular ovals, whereas nuclei of Gata4-positive endoderm cells were more spherical, less flattened, and with a slightly reduced volume. The morphological change was confirmed in the trophectoderm and primitive endoderm lineages of E4.5 blastocysts, compared to larger and more irregularly shaped of the nuclei of the inner cell mass. We established ES cells genetically null for the nuclear lamina proteins lamin A/C or the inner nuclear envelope protein emerin, or compound mutant for both lamin A/C and emerin. ES cells deficient in lamin A/C differentiated to endoderm but less efficiently, and the nuclei remained flattened and failed to condense. The size and shape of emerin-deficient nuclei also remained uncondensed after treatment with RA. The emerin/lamin A/C double knockout ES cells failed to differentiate to endoderm cells, though the nuclei condensed but retained a generally flattened ellipsoid shape. Additionally, ES cells deficient for lamin A/C and/or emerin had compromised ability to undergo endoderm differentiation, where the differentiating cells often exhibited coexpression of pluripotent and differentiation markers, such as Oct3/4 and Gata4, respectively, indicating an infidelity of gene regulation.

**Conclusions:**

The results suggest that changes in nuclear size and shape, which are mediated by nuclear envelope structural proteins lamin A/C and/or emerin, also impact gene regulation and lineage differentiation in early embryos. Nevertheless, mice lacking both lamin A/C and emerin were born at the expected frequency, indicating their embryonic development is completed despite the observed protein deficiency.

**Electronic supplementary material:**

The online version of this article (doi:10.1186/s12860-017-0125-0) contains supplementary material, which is available to authorized users.

## Background

The defining hallmark of eukaryotic cells is the nucleus, which segregates the genetic material, DNA, from diverse cellular and metabolic activities in the cytoplasm. The shape and size of a nucleus are particular to a cell type, function, and cell location [1–3]. Nuclei in columnar shaped cells are elongated along the cell axis, whereas cuboidal cells typically contain rounded nuclei [4]. Nuclear shape can change with age [4], and abnormalities in nuclear morphology serve as indicators of diseases, especially cancer [5–9]. Cancer diagnosis and prognosis frequently rely on the increased size and the degree of irregularity of the nuclei to stage the malignancies [9]. Changes in the nuclear size and shape also associate with cell differentiation and development [8, 10].

The nucleus is bound by a double membrane, or envelope, that consists of the outer nuclear membrane (ONM) and inner nuclear membrane (INM), underlined by a matrix of lamina intermediate filaments and associated proteins [11, 12]. The INM and ONM are compositionally and functionally distinct [13, 14], but connected via nuclear pores and intermembrane protein associations and binding [15]. The nuclear lamin protein network is thought to have an important role in modulating nuclear morphology [11, 16], by increasing the stiffness of the nuclear envelope [17], which can affect nuclear transport [17–20], and likely gene expression [17, 21–23]. The lamin proteins are classified into two families: A-type lamins and B-type lamins [16]. Although the two families share a common basic structure, lamin B proteins appear to be constitutively found in nucleated cells; A-type lamins have increased expression associated with cell differentiation [16, 24, 25].

Changes in the cell and nuclear volume that occur during the development of pre-implantation mouse blastocysts have been previously documented [26]. A reduced physical plasticity of the nucleus [17] and the narrowing of space between the INM and ONM [27] have been noted during the differentiation of ES cells in culture. Expression of nuclear lamin and associated structural proteins is thought to facilitate the structural changes, which can impact chromatin organization and gene expression in ES cell differentiation [28–31]. However, several factors may influence nuclear size and shape, including cytoplasmic and extracellular structures, cytoskeletal tension transduced from the extracellular matrix, and structural components of the nuclear envelope [11, 32–35].

We previously reported that lamin A and its shorter splice isoform lamin C, as well as a number of lamin-associated nuclear envelope structural proteins, were upregulated when murine ES cells differentiated to primitive endoderm cells [27]. In contrast, lamin B levels did not differ significantly between undifferentiated and differentiated cells. We found that the nuclear structure of ES cells undergoes morphological change concomitant with differentiation [27], in particular, the space between the INM and ONM diminishes upon differentiation, and an increased nesprin-1 expression was shown to be the cause of the nuclear envelope structural changes. In the current study, we further examined the morphological changes that accompany differentiation of pluripotent mouse embryonic cells to primitive endoderm-like cells. Specifically, we generated ES cells lacking lamin A/C and/or emerin and tested the importance of these nuclear envelope structural proteins in facilitating the nuclear shape changes during differentiation.

## Results

### Nuclear shape change during embryonic stem cell differentiation in culture

**Fig. 1:**
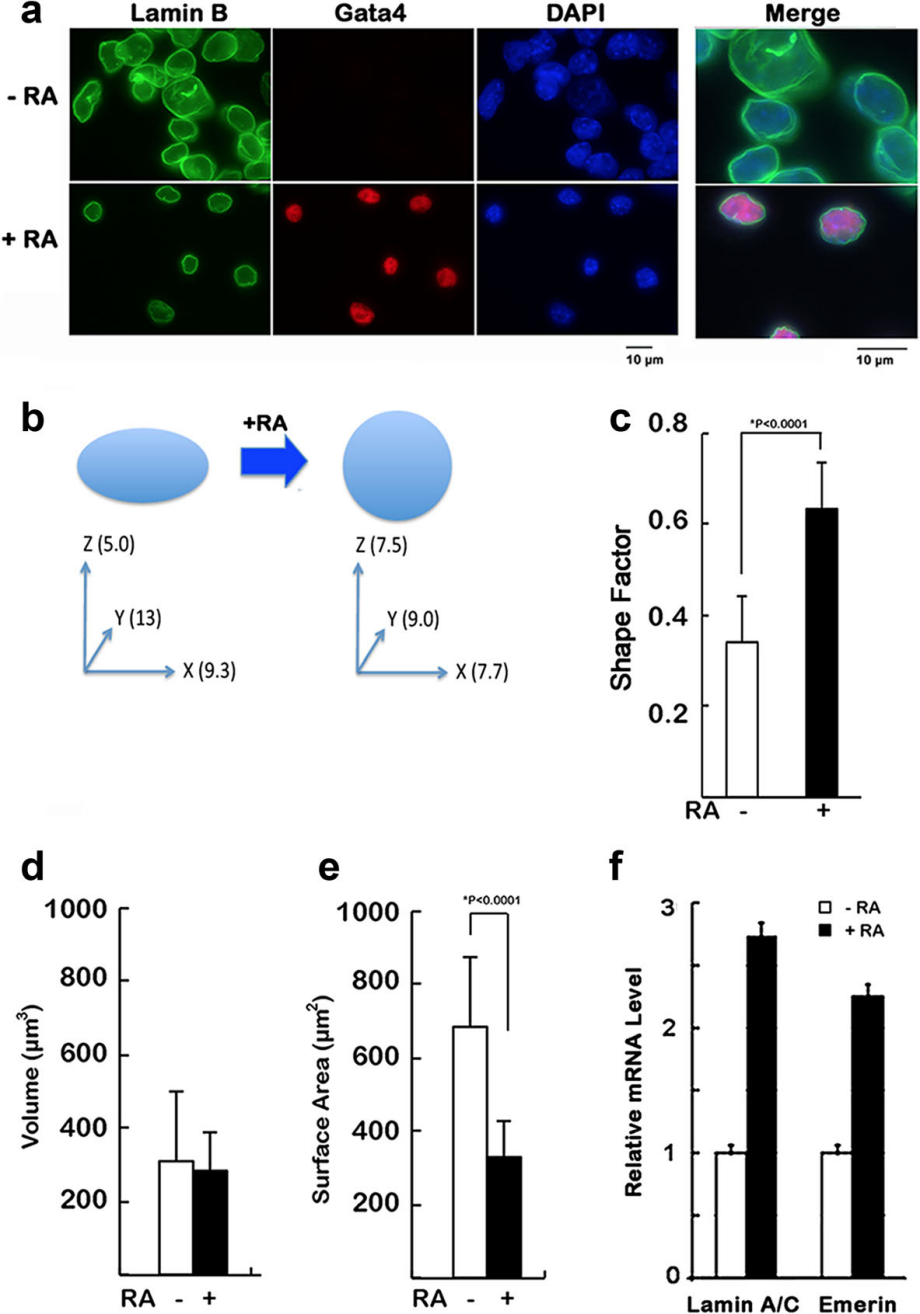
Nuclear shape change during mouse embryonic stem cell differentiation in culture. ES cells in culture were treated for 4 days with or without 1 μM retinoic acid (RA). **a** Confocal immunofluorescence microscopy was performed for immunostaining of lamin B to determine nuclear periphery and Gata4 to indicate differentiated cells, and counterstaining for DNA with DAPI. **b** The change in nuclear shape in the differentiation of ES cells into primitive endoderm is illustrated. The dimensions are indicated as mean values. Optical sectioning of the cells was performed and the z-stacks were analyzed using the Volocity 3D imaging software (Perkins Elmer) to determine nuclear dimensions. **c** Shape factors were calculated based on the average of 50 nuclei analyzed. The difference is statistically significant (*P* < 0.0001). Nuclear volume (**d**) and nuclear surface area (**e**) were calculated. The change in surface area is statistically significant (*P* < 0.0001). **f** mRNA levels of lamin A/C and emerin were determined in triplicate by qRT-PCR using GAPDH for normalization. The relative expression levels are presented as average and standard deviation with the expression in undifferentiated (−**RA**) ES cells defined as “1”

Our initial motivation for the current study was prompted by observations of a striking nuclear size change observed during the differentiation of ES cells in culture. Using staining of lamin B to demarcate the nuclear periphery, and Oct3/4 as marker for undifferentiated ES and Gata4 for differentiated cells, we sought to document the nuclear shape change accompanying retinoic acid-induced ES cell differentiation in culture (Fig. 1). Undifferentiated ES cells characteristically grow as colonies, or clusters of cells, and the nuclei are generally irregularly shaped ovoids (Fig. 1a, upper panel). Treatment of the ES cells with all-*trans* retinoic acid (RA) for 4 days induced the cells to differentiate to Gata4-positive primitive endoderm cells, and caused an obvious reduction in the 2-dimensional size of the nuclei (Fig. 1a, lower panel). Gata4-positive nuclei appear noticeably smaller and rounder than the undifferentiated ES cells (Fig. 1). Optical sectioning through the cells by confocal microscopy was used to determine the nuclear shape and volume (Fig. 1b). We designated the *y*-dimension as the longest and the *x-*dimension as the shorter axis in the same horizontal focal plane, and the *z*-dimension as the vertical height. The undifferentiated nuclei were relatively flat (*z* = 5.0 ± 0.95 μm) and shaped like a lozenge (Fig. 1b, left panel), and resembled an oblate ellipsoid. Differentiation increased the height of the nuclei, with an average *z*-depth that equaled 7.5 ± 1.1 μm (*P* < 0.0001) (Fig. 1b, right panel). The undifferentiated ES cells had longer *x*- and *y*- dimensions, and shorter *z*-dimensions, while all dimensions approximated near equal diameters in the nuclei of the differentiated cells, such that the nucleus resembled a sphere. To assess more quantitatively the degree of roundness of nuclear shape, we calculated the shape factor for each nucleus, which measures both the relative roundness and roughness of the shape [36]. A perfectly smooth sphere has a shape factor that equals “1.0”; conversely, the more elliptical and convoluted outlines have smaller shape factor values, approaching the lower limit “0” [37]. The undifferentiated ES cells had an average shape factor of about 0.34, while the shape factor of differentiated ES cells was approximately double, indicating that differentiation caused a greater prevalence of more spherical, smooth nuclei (Fig. 1c).

In addition, using Volocity 3D imaging software to calculate approximate volumes of the nuclei as described previously [38], we found that volume of the differentiated nuclei did not change from the control, undifferentiated nuclei; however, the surface area of the differentiated nuclei decreased by approximately 50% (Fig. 1d). Thus, we documented that in culture, ES cells undergo a nuclear shape change from a flat oblate ellipsoid to a more spherical pattern, with minor change in volume (Fig. 1e).

Previously we have found that the expression of several nuclear structural proteins is increased when ES cells are induced to differentiate [27]. Since lamin A/C and emerin proteins have a strong influence on nuclear shape [37, 39], we examined the change in their expression associated with ES cell differentiation. Using qRT-PCR, lamin A/C and emerin were found to be already present in ES cells, and RA-induced differentiation led to a 2–3 fold increase in both lamin A/C and emerin mRNA (Fig. 1f).

### Distinctive nuclear shapes of early lineages in blastocysts

**Fig. 2:**
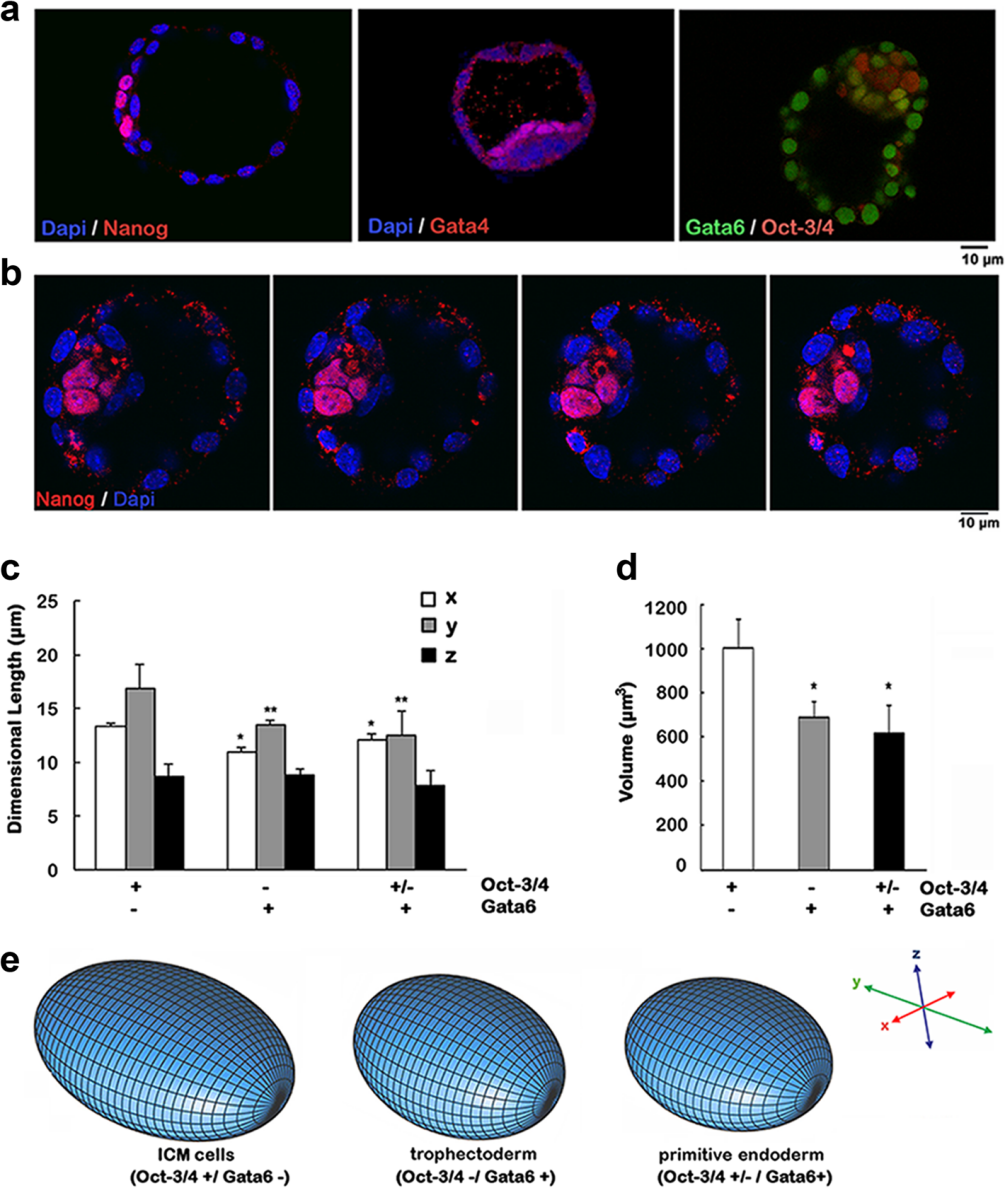
Diverse nuclear shapes of early embryonic lineages in mouse blastocysts. A total of 16 E4.5 blastocysts were analyzed for expression of Nanog, Oct-3/4, Gata4, and Gata6 by confocal immunofluorescence microscopy. **a** Examples of immunostaining of representative blastocysts. Nanog and Oct-3/4 highlight the ICM; Gata4 staining indicates the nuclei of the primitive endoderm; Gata6 marks both the trophectoderm and the primitive endoderm. **b** Sequential confocal sections taken of a representative blastocyst show the larger and somewhat irregular shape of the nuclei of cells of the ICM, which immunostain positively for Nanog. **c** The dimensions of the nuclei of cells of pluripotent ICM (positive immunostaining for Oct3/4 +, negative for Gata6 -), primitive endoderm (GATA4 +), and trophectoderm (GATA6 +, GATA4 -) were measured using confocal sectioning of the blastocysts. The values are presented as mean ± s.d. The changes in nuclear dimensions between undifferentiated (ICM) and differentiated cells were found to be statistically significant for the *x*- (*) and *y*-dimensions (**) (*P* < 0.0001). **d** Nuclear volume for the cells was calculated. Statistical significance (*P < 0.0001) was found between undifferentiated (Oct-3/4-positive and Gata6-negative immunostaining) compared to differentiated cells, both primitive endoderm and trophectoderm. **e** Schematic 3D models of the nuclei of the cells in the E4.5 embryos were constructed using the metrics, as described in “Methods”

To determine if the observed nuclear shape changes in cultured ES cells occurs in embryos, we analyzed the nuclear shape changes during lineage commitment of early stage mouse embryos. The changes in volume and nucleo-cytoplasmic ratio in pre-implantation embryos up to primitive endoderm have been observed and described [26]. We examined a later stage, the E4.5 mouse embryo, for differences in the nuclear shape and volume of cells comprising the trophectoderm, primitive endoderm, and pluripotent cells of the inner cell mass (Fig. 2a-c, Table 1), using Gata6, Gata4, Nanog and Oct3/4 as markers, respectively.

**Table 1:** Nuclear measurements in blastocysts. Confocal imaging analysis was used to determine the *x*-, *y*-, and *z*-dimensions of nuclei in E4.5 blastocysts. The volumes of the nuclei were calculated from the measurements, as described in Methods

Blastocyst Nuclei	Oct4 + / Gata6 – ICM cells	Oct4 - / Gata6 + Trophectoderm	Oct4 +/− / Gata6 +Primitive endoderm
*(μm)*	*Mean ± S.D.*	*Mean ± S.D.*	*Mean ± S.D.*
*x*- dimension	13.3 ± 0.27	10.98 ± 0.41	11.99 ± 0.56
*y*- dimension	16.8 ± 2.36	13.5 ± 0.43	12.5 ± 2.25
*z*- dimension	8.73 ± 1.12	8.78 ± 0.55	7.79 ± 1.51
Volume (μm^3^)	1001 ± 129	688 ± 73	616 ± 126

The ICM cells (Nanog and Oct3/4 positive) had larger, flatter and somewhat irregularly shaped nuclei compared to the differentiated endoderm (Gata4, Gata6, and weakly Oct3/4 positive) and trophectoderm cells (Gata6 positive), which appeared as uniformly shaped condensed ovoids in serial confocal sections (Fig. 2b). Persistence of Oct3/4 in primitive endoderm nuclei agrees with previous reports that Oct-3/4 expression may be involved in triggering primitive endoderm differentiation [40], whereas suppression of Nanog expression indicates *bona fide* loss of pluripotency [41]. Additionally, the volumes of the differentiated nuclei found in both the trophectoderm and endoderm were reduced approximately 40% from the undifferentiated nuclei of the ICM (Fig. 2d).

Thus, nuclear shape and volume changes in the early lineages of the embryos are distinct from those of ES cell differentiation in culture. Nevertheless, the occurrence of flat to round nuclear shape change in differentiation of embryonic cells is consistent in both embryos and cultured cells (Fig. 2e).

### Lamin A/C and/or emerin impact lineage differentiation of embryonic stem cells

Expression of nuclear envelope structural proteins is expected to impact nuclear shape, and we sought to determine if nuclear lamin A/C and its anchoring protein emerin mediate nuclear shape change during ES cell differentiation. We set out to generate panels of ES cells deficient of either lamin A/C (*lmna* gene) and/or emerin (*emd* gene) from established knockout mice.

**Fig. 3:**
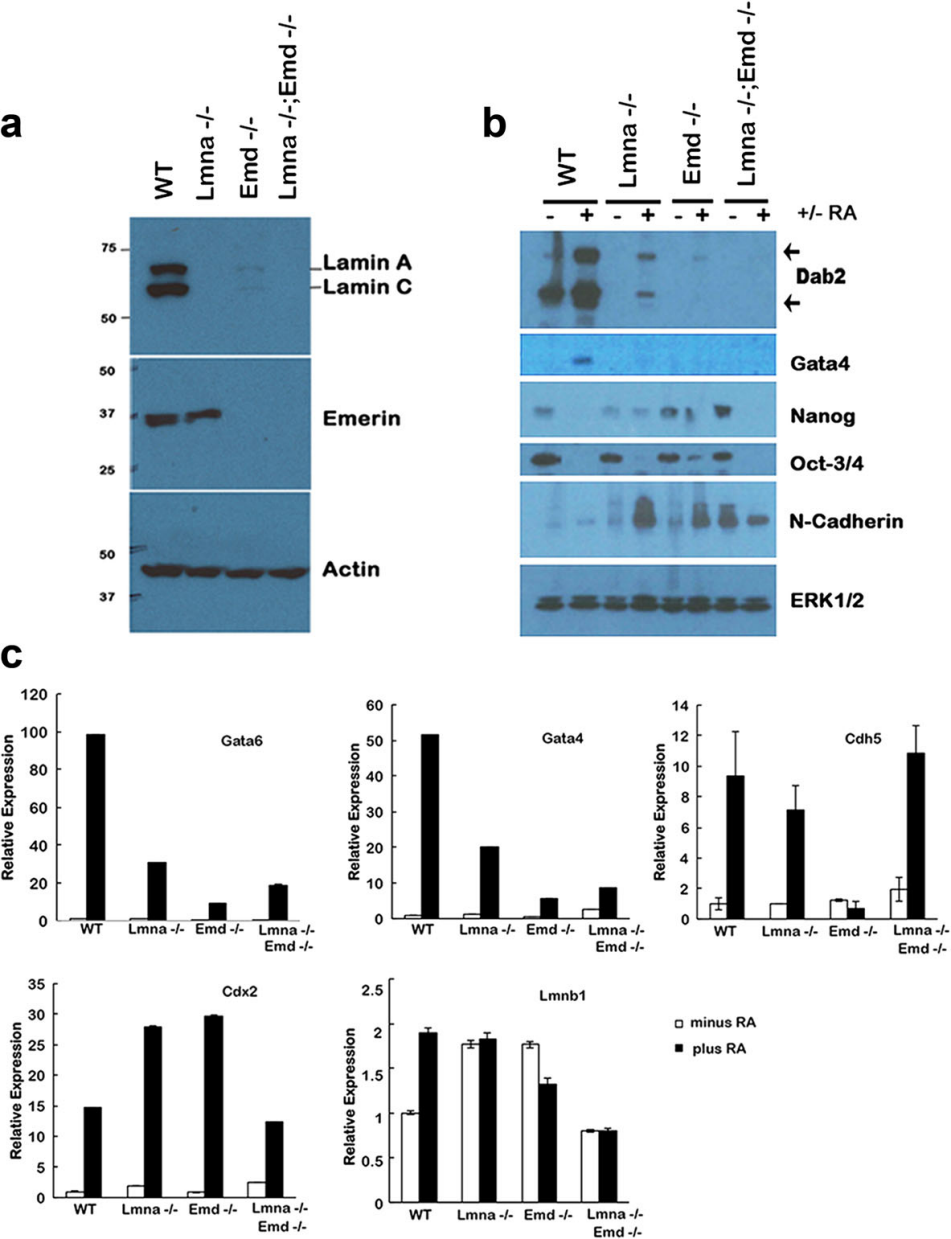
Reduced primitive endoderm differentiation of ES cells deficient of lamin A/C and/or emerin. **a** A Western blot shows the absence of lamin A/C and/or emerin proteins in ES cell lines with *lmna* (−/−) and/or *emd* (−/−) genotypes. Cells were incubated for 4 days with 1 μM RA, and cell lysates were prepared. **b** Wild type, *lmna* (−/−) and/or *emd* (−/−) ES cells were treated with or without RA for 4 days. The cell lysates were analyzed by Western blot. Protein loading was normalized to total (pan) ERK1/2. **c** The undifferentiated and differentiated cells were analyzed by qRT-PCR for expression of various markers. Values for each sample were normalized to respective GAPDH levels and plotted relative to control, untreated wild type (wt), which is defined as “1”

**Fig. 4:**
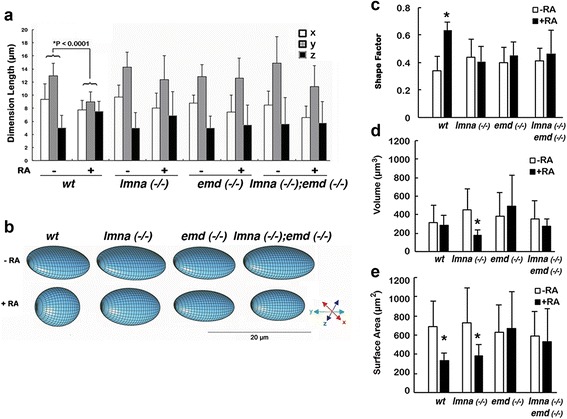
Lamin A/C and/or emerin mediate nuclear shape changes in embryonic stem cell differentiation. ES cells of wild type (wt), *lmna* (−/−), *emd* (−/−), and *lmna* (−/−);*emd* (−/−) ES cells were treated with 1 μM RA for 4 days. Immunostainings for Gata4 and Oct3/4 to indicate differentiated or undifferentiated cells, and lamin B to mark nuclear outline, were imaged by confocal microscopy, followed by analysis using Volocity 3D imaging software. The measurements obtained (Table 2) were used to analyze nuclear shape. **a** The nuclear dimensions are plotted as mean ± s.d. Only the wild type ES cells show statistically significant (*P < 0.0005) changes in nuclear shape between differentiated and undifferentiated states. **b** The changes in nuclear shape were modeled for illustrative purposes. Shape factors (**c**), nuclear volume (**d**), and nuclear surface area (**e**) were calculated based upon the quantified dimensions

From harvested blastocysts, we produced 4 to 7 clones of each genotype: wild type (*wt*) and heterozygous, *lmna* (−/−), *emd* (−/−), and *lmna* (−/−);*emd* (−/−) ES cells lines. Initial tests indicated the phenotypes of heterozygous cells were indistinguishable from null cells, and thus 3 lines each of *wt*, *lmna* (−/−), *emd* (−/−), and *lmna* (−/−);*emd* (−/−) ES cells were expanded and used for subsequent analyses. Western blotting indicates the complete absence of lamin A/C in *lmna* (−/−) and *lmna* (−/−);*emd* (−/−) ES cells, and emerin in *emd* (−/−) and *lmna* (−/−);*emd* (−/−) lines (Fig. 3a). Interestingly, lamin A/C proteins were greatly reduced (observable only in higher exposures of the Western blot) in emerin-deficient ES cells [See Additional files 1 and 2]. However, deletion of *lmna* had little influence on emerin protein level (Fig. 3a). In the undifferentiated stage, the ES clones (*wt*, *lmna* (−/−), *emd* (−/−), and *lmna* (−/−); *emd* (−/−)) showed no statistically significant differences in nuclear volume, surface area, or contour factor (Fig. 4, Table 2).

**Table 2:** Measurements of nuclear parameters in wild type and mutant ES cells. Embryonic stem cells were plated on gelatinized tissue culture dishes and treated with or without RA for 4 days

ES Nuclei	Wild type		*lmna* (−/−)		*emd* (−/−)		*lmna* (−/−);*emd* (−/−)	
mean *(μm ± s.d.)*	- RA *(n = 57)*	+ RA *(n = 65)*	- RA (*n = 50)*	+ RA *(n = 34)*	- RA *(n = 49)*	+ RA *(n = 22)*	- RA *(n = 48)*	+ RA *(n = 34)*
*x*- dimension	9.33 ±2.34	7.73 ±1.43	9.67 ±1.9	8.01 ±2.28	8.75 ±1.3	7.38 ±2.62	8.48 ±2.11	6.60 ±1.72
*y*- dimension	12.94 ±1.90	8.97 ±1.53	14.27 ±2.32	12.36 ±3.37	12.80 ±1.83	12.60 ±3.03	14.87 ±4.04	11.27 ±3.22
*z*- dimension	4.97 ±0.95	7.50 ±1.11	4.95 ±0.88	6.88 ±1.62	4.99 ±0.94	5.42 ±1.09	5.59 ±0.90	5.753 ±1.2
Shape factor	0.34	0.62	0.42	0.40	0.40	0.43	0.42	0.44
Volume (μm^3^)	333 ± 109	271 ±83	368 ±89	313 ±128	297 ±101	289 ±178	370 ±132	223 ±102
*P* value	*P < 0.0005		*P < 0.05		*P* = 0.810		*P < 0.0001	

Measurements were made as described in Methods for wild type, *lmna* (−/−), and/or *emd* (−/−) cells. For the undifferentiated stage (− RA), values were determined in Oct3/4-positive cells. In differentiated cells (+ RA), only Gata4-positive nuclei were measured. Since no Gata4-positive nuclei were detected in *lmna* (−/−);*emd* (−/−) cells following treatment with RA, Oct3/4-negative (differentiated) nuclei were measured. The values were mean of the indicated number of nuclei analyzed with standard derivation shown. Statistically significant differences in nuclear volumes are indicated by *P

In multiple experiments and in all clones tested using identical differentiation conditions with retinoic acid treatment, *lmna* (−/−), *emd* (−/−), and *lmna* (−/−);*emd* (−/−) ES cells showed a marked reduction in primitive endoderm differentiation, as indicated by the induction of Gata4 and Dab2 proteins (Fig. 3b). Nevertheless, cells lacking lamin A/C and/or emerin lost pluripotency following treatment with retinoic acid. These cells gained the expression of N-cadherin, a marker for neuronal and mesodermal differentiation. This differentiation to a neuronal lineage is consistent with low levels of lamin A found in the brain, which is regulated in normal cells by miR-9, a brain-specific microRNA [42, 43].

Quantitation of mRNA using qRT-PCR indicated the transcription of primitive endoderm markers Gata4 and Gata6 was reduced in cells deficient of lamin A/C and/or emerin (Fig. 3c). Differentiation toward the trophectoderm lineage, indicated by Cdx2 expression, was increased in the absence of lamin A/C or emerin. Expression of Cdh5 (encoding VE-Cadherin), a marker for endothelial lineage, was reduced only in emerin deficient cells. Expression of lamin B (Lmnb1) was increased in the absence of lamin A/C or emerin, likely as a mechanism of compensation for the loss or reduction of lamin A/C.

Thus, we conclude that lamin A/C and emerin are required for efficient primitive endoderm differentiation of ES cells in culture, and the absence of lamin A/C and emerin alters lineage differentiation induced by retinoic acid.

### Lamin A/C and/or emerin mediate nuclear shape changes in embryonic stem cell differentiation

We used confocal microscopy and lamin B1 immunostaining to analyze nuclear shape changes in the differentiation of the wild type and mutant ES cells (Fig. 4, Table 2). Nuclei from undifferentiated mutant ES cells typically were shaped as oblate spheroids, or flattened ellipsoid structures, and similar in shape to undifferentiated wild type nuclei (Fig. 4a). No untreated cells exhibited Gata4-positive nuclei; rather most cells were Oct3/4-positive. Treatment of the mutant cells with RA failed to trigger a similar shape change that occurred in wild type ES cells (Fig. 4a). *lmna* (−/−) ES cells often had irregularly shaped nuclei, even in the Gata4-positive, primitive differentiated cells (Fig. 4b), and the shape factor was unchanged with respect to the undifferentiated cells (Fig. 4c). We also observed only in *lmna* (−/−) and not in *emd* (−/−) cells a particular reduction of nuclear volume and surface area. The extent of nuclear shape irregularity in *emd* (−/−) ES cells was not as prominent as in *lmna* (−/−) cells (Fig. 4b). Nevertheless, primitive endoderm differentiation of *emd* (−/−) ES cells, like that of *lmna* (−/−), did not lead to an increased shape factor value as seen in wild type ES cells (Fig. 4c).

Thus, either lamin A/C and/or emerin are required for nuclear shape change associated with ES cell primitive endoderm differentiation (loss of pluripotency in the case of *lmna* (−/−) and *emd* (−/−) ES cells). Additionally, the observation suggests that emerin but not lamin A/C plays a specific role in mediating the reduction of nuclear volume during differentiation.

### Gene expression infidelity during differentiation of ES cells null for lamin A/C and/or emerin

**Fig. 5:**
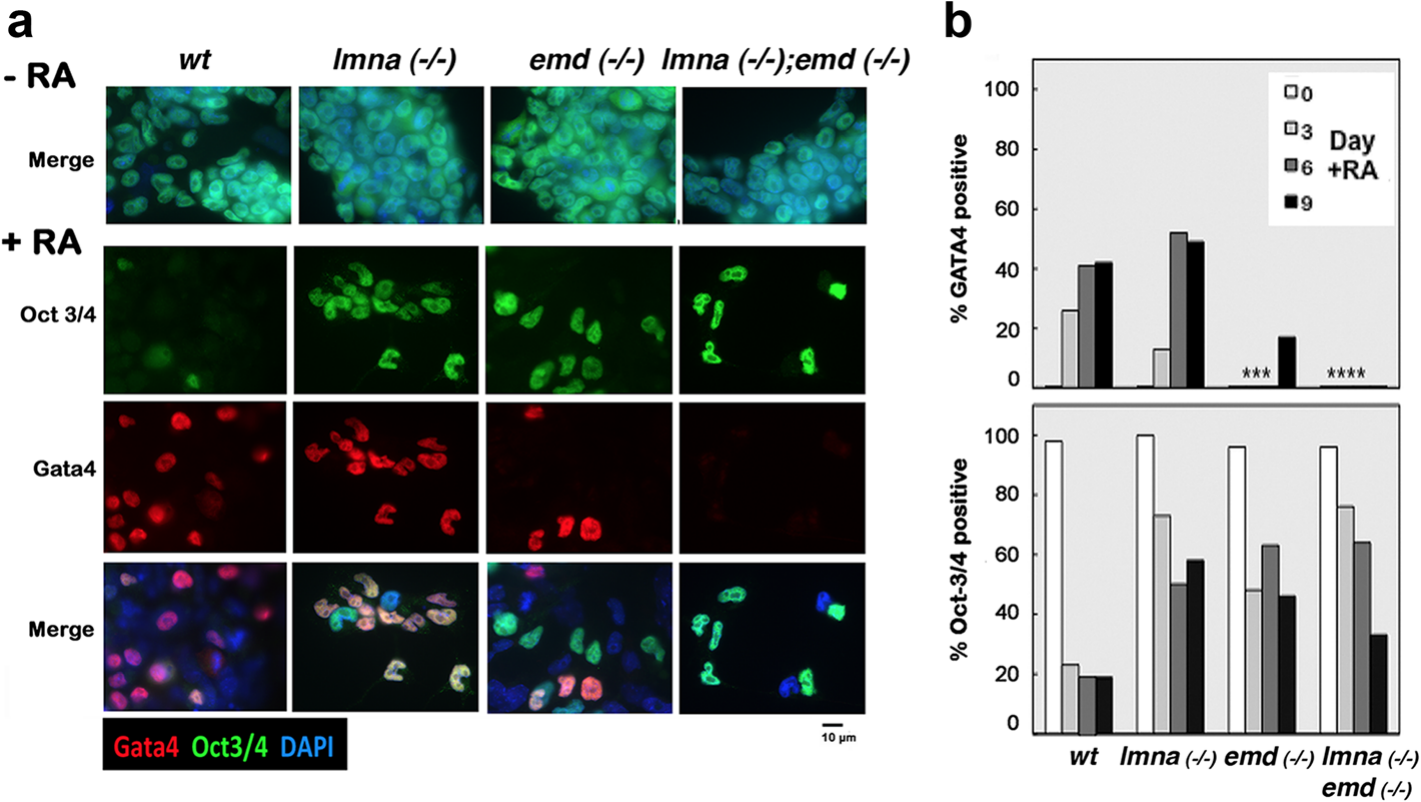
Lamin A/C- and/or emerin-deficient ES cells show gene expression defects during differentiation. Wild type (wt), *lmna* (−/−) and/or *emd* (−/−) ES cells were treated with or without RA, and analyzed by confocal immunofluorescence microscopy for Oct3/4 and Gata4. **a** Representative examples of ES cells treated with or without retinoic acid are shown. **b** Oct3/4 and Gata4 expression was quantitated in the ES cells treated with 1 μM RA for 0, 3, 6, and 9 days. About 200 nuclei in each group were scored for Gata4 and Oct-3/4 staining, expressed as the percentage of the total number of nuclei. Gata4-positive nuclei were few (2 to 18%) in *emd* (−/−) cells (***) and undetectable in the *lmna* (−/−);*emd* (−/−) cells (****)

Because ES cells deficient in lamin A/C and/or emerin were impeded to undergo retinoic acid-induced primitive endoderm differentiation in culture, we used immunofluorescence microscopy to assess the expression of Oct3/4 and Gata4, and quantitate loss of pluripotency and differentiation towards primitive endoderm, respectively (Fig. 5). Initially, the undifferentiated wild type and mutant ES cells were predominantly Oct3/4 positive (Fig. 5a). Wild type ES cells responded to retinoic acid and rapidly differentiated: Gata4-positive cells increased and Oct3/4 expression was lost (Fig. 5b). ES cells lacking lamin A/C were compromised in their differentiation to Gata4-positive endoderm cells, while the ability of the emerin-deficient cells to differentiation was almost totally absent (Fig. 5b). Nevertheless, the cells deficient in either lamin A/C or emerin lost pluripotency, indicating differentiation into lineages other than primitive endoderm, though at a reduced degree compared to wild type cells (Fig. 5b). Particularly, differentiated *lmna* (−/−) and *emd* (−/−) ES cells often simultaneously expressed Gata4, a differentiation marker, and Oct3/4, an indicator of undifferentiated, pluripotent state (Fig. 5a), indicating gene expression infidelity. These observations suggest that lamin A/C and/or emerin deficient ES cells are defective in the regulation of gene expression during differentiation.

**Fig. 6:**
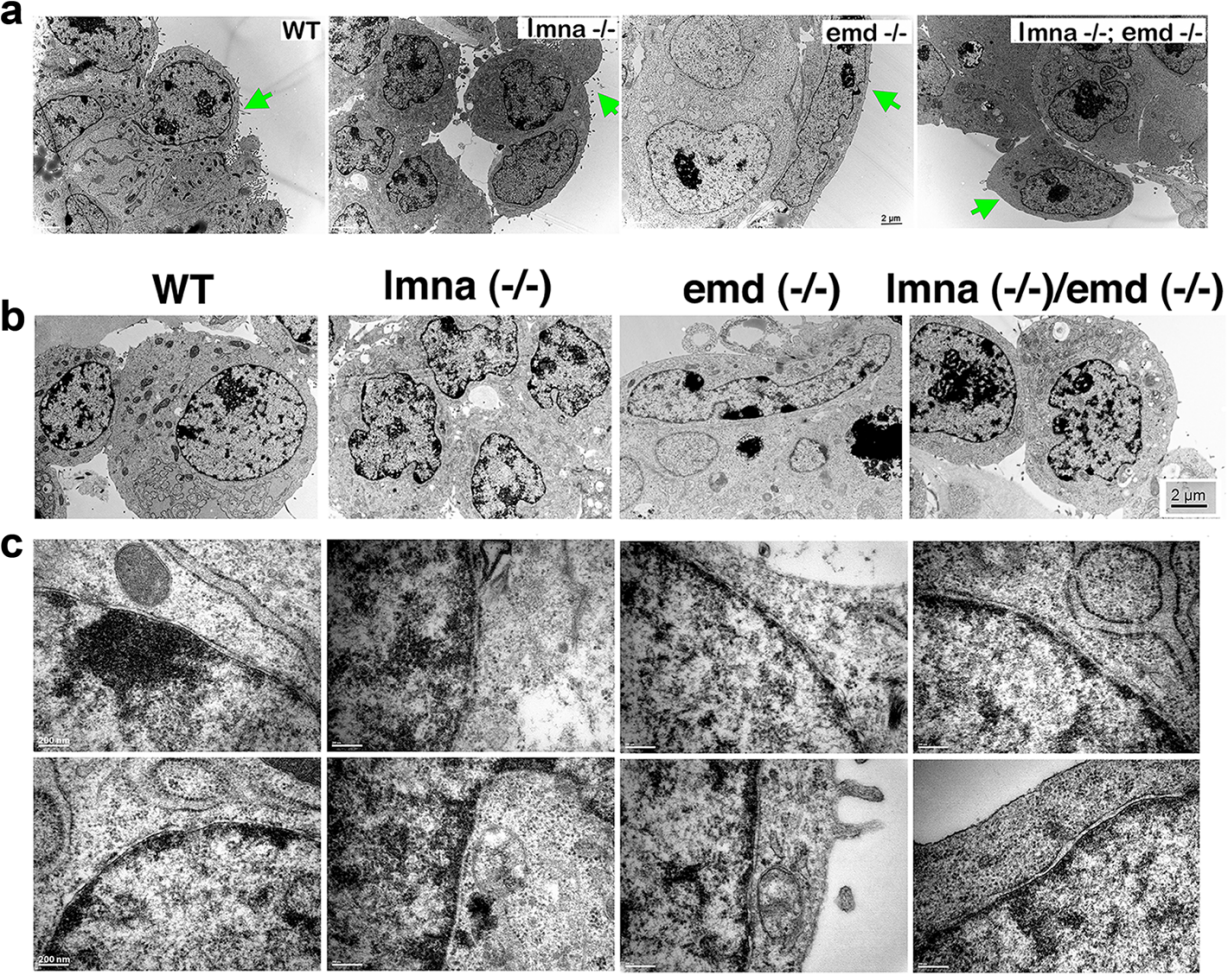
Cell surface morphology and chromatin organization in wild type and mutant embryoid bodies. Wild type and mutant ES cells were cultured in suspension culture as embryoid bodies and treated with 1 μM RA for 5 days. Embryoid bodies were analyzed by transmission electron microscopy. **a** Representative images of the outer differentiated layer of cells. Arrows mark microvilli-containing endoderm cells. **b** Representative low magnification images of outer layer, presumably differentiated cells from wild type and mutant embryoid bodies. **c** Higher magnification images from two different nuclei for each cell line show the positioning of electron dense heterochromatin in relation to the inner nuclear membrane

Since the nuclear lamina likely influences gene regulation by binding to and modulating chromatin organization, we examined by transmission electron microscopy (EM) the differentiated chromatin phenotypes of wild type and mutant ES cells. The ES cells were allowed to aggregate into embryoid bodies in suspension and exposed to 1 μM RA for 5 days. This treatment causes differentiation of an endoderm outer layer, characterized by the presence of microvilli easily detectable in EM images (Fig. 6a). Wild type differentiated cells had numerous microvilli, and *lmna* (−/−) also exhibited microvilli from the surface layer of cells, which supports other evidence that the *lmna* (−/−) cells did actually differentiate to endoderm cells. Few or no microvilli were found on embryoid bodies from *emd* (−/−) and *lmna* (−/−)*;emd* (−/−) cells. Gross examination of chromatin in the presumably differentiated cells did not reveal a striking distinction between the mutant and wild type cells (Fig. 6b). However, higher magnification showed that heterochromatin was organized in compact areas along the inner nuclear envelope in wild type cells; in *lmna* (−/−) cells, heterochromatin was more loosely associated with the nuclear envelope (Fig. 6c). *emd* (−/−) cells had a thin ring of heterochromatin associated with the nuclear envelope and small electron-dense clusters near offset from the envelope. The double knockout cells exhibited both condensed chromatin localized along the inner nuclear envelope as well as more loosely associated clusters. In addition, the irregularity of the nuclear shape is obvious in the mutant cells (Fig. 6a, b).

### Loss of both lamin A/C and emerin does not impair embryonic development in mice

**Fig. 7:**
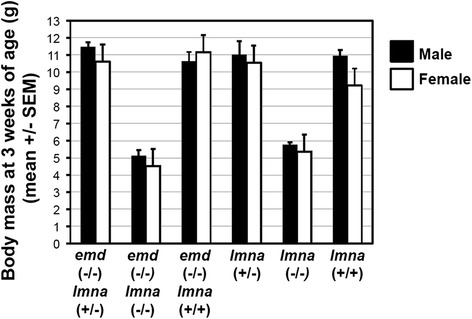
Loss of both lamin A/C and emerin does not impair embryonic development in mice. *lmna* (+/−) and *emd* (−/−) mice were crossed to establish colonies. Using *lmna* (+/−); *emd* (−/−) parents, matings were performed and progenies at 3 weeks of age were genotyped by PCR of tail tissues. The weights were determined for each mouse, and means +/− SEM are presented. Genotypes and weights of progenies from mating of *lmna* (+/−) mice were determined for comparison

Although lamin A/C and emerin appear to play roles in nuclear morphogenesis and regulation of lineage differentiation in early embryos, neither *lmna* or *emd* gene deletion impaired the development of mouse embryos [39, 43]. We further tested if deletion of both *lmna* and *emd* gene simultaneously would impact mouse embryonic development. Using *lmna* (+/−);*emd* (−/−) parents, we performed matings and analyzed the genotypes of the progenies obtained. Previously in the process of generating mutant ES cell lines, we observed the presence of *lmna* (−/−);*emd* (−/−) blastocysts. Somewhat surprisingly, *lmna* (−/−);*emd* (−/−) mice were born at a roughly expected frequency (Table 3), indicating both lamin A/C and emerin are dispensable for mouse embryonic development. Lamin A/C null mice are known to be runted or growth-inhibited and die at 6 to 8 weeks of age due to heart defects [39]. We observed that *lmna* (−/−); *emd* (−/−) mice also died around 6 to 8 weeks of age, indistinguishable from *lmna* (−/−) littermates. Additionally, measurement of entire body weight as an indication of general hardiness of the mice indicated that wild type, *emd* (−/−) and/or *lmna* (+/−) had similar weights, and *lmna* (−/−);*emd* (−/−) mice had similarly reduced body masses as *lmna* (−/−) animals (Fig. 7).

**Table 3:** Distribution of genotypes in progenies from matings between *emd* (−/−); *lmna* (+/−) parents

Genotype	gender	number	%	Total number	Total %
*emd* (−/−);*lmna* (+/+)	male	*n* = 10	13%	*n* = 18	23%
	female	*n* = 8	10%		
*emd* (−/−);*lmna* (+/−)	male	*n* = 24	31%	*n* = 44	57%
	female	*n* = 20	26%		
*emd* (−/−);*lmna* (−/−)	male	*n* = 9	12%	*n* = 15	19%
	female	*n* = 6	8%

Thus, deletion of both *lmna* and *emd* does not impair embryonic development, and the double knockout has similar post-developmental defects as *lmna* deletion alone, suggesting that lamin A/C is able to function without the presence of emerin, and that the cellular functions of lamin A/C and emerin are largely redundant.

## Discussion

In the current study, we showed that the nuclei of ES cells change from a flat oblate ellipsoid to a sphere shape during differentiation in culture, and that both lamin A/C and/or emerin are required for the shape change. Additionally, we found that ES cells lacking lamin A/C and/or emerin had altered ability to undergo lineage commitment and gene expression.

Emerin knockout ES cells also have greatly reduced lamin A/C protein, but lamin A/C deletion has little effect on emerin level. Thus, at the early embryonic stages, lamin A/C function largely depends on binding to emerin. Consistently, emerin knockout ES cells have a more severe phenotype of inability to undergo primitive endoderm differentiation than lamin A/C-deficient cells. However, emerin knockout mice have no noticeable phenotype and survive into adulthood and breed [43], whereas lamin A/C knockout mice die at 6 to 8 weeks of age [39, 44]. Moreover, likely in the mature mouse, the lamin A/C protein level is not significantly affected by the deletion of emerin, as was shown previously [44]. Lamin A/C can apparently function in later stages in an emerin-independent manner. Consistently, double knockout mice have similar postnatal defects as *lmna* deletion alone. Likely, additional lamin A/C binding proteins that are expressed later in development are able to substitute for the function of emerin, at the least in mice. Somewhat surprisingly, embryos deficient in either A-type or B-type lamins survive development [43, 45]. The idea has been posited that although the lamin proteins have specialized functions in differentiated cells, they behave more non-specifically in embryos, such that compensation by other proteins allows the embryos to survive until birth [46]. It may be the accumulation of defects, and especially the possible deformation of nuclear structure due to physical force in postnatal stages that may contribute to the pathologies due to the loss of lamin A/C and emerin function.

This finding is especially intriguing given that lamin A/C are linked to several diseases, including the cardiomyopathy, muscular dystrophy, and peripheral neuropathy that characterize and cause death in the lmna (−/−) mice before 2 months of age [39, 44], as well as Hutchison-Gilford progeria syndrome, adult-onset partial lipodystrophy, and mandibuloacral dysplasia [47, 48]. Mutations in *EMD* in humans cause Emery-Dreifuss muscular dystrophy [49], characterized by skeletal muscle wasting and cardiomyopathy, and an autosomal dominant form is linked to mutations in *LMNA* [50]. The deletion of lmna in mice causes a similar phenotype to Emery-Dreifuss muscular dystrophy. Because lamin A/C interacts with emerin and mediates its localization to the inner nuclear membrane, emerin and lamin A/C act on the same pathway to cause the pathologies. It is possible in some differentiated cells, defects in emerin may lead to reduction in lamin A/C proteins, which contribute to the disease pathology.

In culture, both lamin A/C and/or emerin-deficient ES cells are unable to facilitate the change of nuclear shape during differentiation. However, lamin A/C and emerin are already expressed in ES cells prior to differentiation, and are increased only 2- to 3-fold [27]. Thus, likely induction of a differentiation specific protein(s) is a true trigger in nuclear shape change. We have noted previously that a number of lamin-associated proteins are upregulated during differentiation of ES cells induced by RA [27]; how these protein associations and functions are altered by lamin A/C and emerin deletion and by changes in nuclear structure and shape will be interesting to determine. Possibly, this lamin A/C or emerin binding protein(s) alters the assembly of the lamin A/C-emerin lamina scaffold, and increases the stiffness of the nuclear envelope structure. Similar phenotypes are presented in lamin A/C or emerin null ES cells, though some subtle differences are present. The ES cells deficient of lamin A/C but not emerin reduce nuclear volume and surface area upon differentiation, suggesting emerin plays a role in controlling nuclear volume. Induced expression of cdh5 upon differentiation is impaired in emerin-deficient but not in lamin A/C-deficient cells.

The absence of lamin A/C and/or emerin impacts fidelity of gene expression during the differentiation of ES cells. This is likely mediated by the activity of the nuclear lamina in binding of chromatins and modulating the transcriptional activity [51]. In ES cells the chromatin is diffuse as lightly packed euchromatin, which is permissive to transcription, while the chromatin is generally more tightly packed heterochromatin in differentiated cell nuclei [52]. We found subtle differences from wild type in chromatin organization near the periphery of the inner nuclear envelope in the differentiated mutant cells. The role of lamina in controlling gene and nuclear shape in ES cells is dispensable for embryonic development. Nevertheless, the nuclear envelope structural defect leads to lethal pathology in the *lmna* knockout mice at a later stage.

Thus, the study of the nuclear envelope defects of ES cells in culture reveals the potential mechanisms of pathology such as displayed in laminopathies [21, 39, 44, 45, 48] and cancer [9, 53–55].

## Conclusions

Changes in nuclear size and shape, which are mediated by nuclear envelope structural proteins, lamin A/C and/or emerin, also impact gene regulation and lineage differentiation in early embryos. However, mice lacking both lamin A/C and emerin are produced at the expected frequency, indicating their embryonic development is completed despite the observed protein deficiency.

## Methods

### ES cell culture

RW4 mouse ES cells (ATCC) were maintained as previously described [56-58]. Cells were seeded on gelatin-coated tissue culture plates without feeder cells for two days in ES cell medium containing LIF (DMEM, 15% (v/v) FBS, 2 mM L-glutamine, 1X non-essential amino acids, 50 IU/ml penicillin, 50 mg/ml streptomycin, 0.1 mM β-mercaptoethanol, and 1,000 U/ml LIF), then trypsinized and replated on gelatinized plates, and induced with 1 μM all-*trans*-retinoic acid (RA) for at least 4 days, as described previously [57, 58]. Lamin A/C [39], emerin [44], and the lamin A/C-emerin knockout ES cells were isolated from pre-implantation E3.5 day old mouse embryos as described previously [58, 59]. Genotyping for *emd* used the published PCR primer sequences [44]. PCR primers for *lmna* genotyping were: 5’ CAA GTC CCC ATC ACT TGG TT 3’, 5’ CTG TGA CAC TGG AGG CAG AA 3’, and 5’ GCC AGA GGC CAC TTG TGT AG 3’, with wild type lamin A/C found at 314 bp and the mutated allele at 154 bp. Lamin A/C knockout (*lmna* (−/−)) ES cells were generated from an embryo that originated from matings between *lmna* (+/−) mice. Emerin knockout (*lmna* (+/−);*emd* (−/−)) ES cells and LaminA/C-Emerin double knockout (*lmna* (−/−);*emd* (−/y)) ES cells both resulted from embryos that were generated after crossing *lmna* (+/−);*emd* (−/y) with *lmna* (+/−);*emd* (−/−). Mutant ES cells were maintained as described for RW4 wild type ES cells. All tissues were collected from animals euthanized using isoflurane anesthesia overdose, confirmed by cervical dislocation. All animal usage was approved by the University of Miami IACUC and met ethical guidelines.

ES cells were cultured in suspension in non-tissue culture treated petri plates to form cell aggregates, or embryoid bodies [58]. Typically, following trypsinization of adherent cultures, 6 × 10^6^ cells were incubated in 10 ml of ES cell medium lacking LIF with or without 1 μM RA for 5 days. Medium was changed every two days by allowing the embryoid bodies to sediment by gravity and then aspirating the media.

### Indirect immunofluorescence staining of blastocysts

E3.5 day blastocyst embryos were isolated from C57BL/6 J crosses. Blastocysts were flushed from the uterine horns, collected into a 60-mm dish, and incubated overnight in 300 μl of KSOM medium (EMD-Millipore, Billerica, MA), in a 5% CO_2_ incubator at 37 °C, overlaid with mineral oil to limit evaporation. The next day, individual blastocysts (referred to now as at E4.5 stage) were transferred to single wells of a 96-well round bottom plate and fixed in 4% paraformaldehyde plus 5% sucrose and 1% Triton X-100 for 1 h at RT, washed twice in PBS by transferring the blastocysts to new wells, and blocked overnight in 5% BSA at 4 °C. Blastocysts were incubated with the appropriate primary antibodies diluted in 5% BSA for 1 h at RT, washed, and blocked overnight in 2% BSA at 4 °C. Primary antibodies used were: rabbit anti-Nanog (1:300, Abcam, ab80892, Cambridge, MA); rabbit polyclonal anti-Gata4 (1:300, Santa Cruz, sc9053); rabbit polyclonal anti-Gata6 (1:1000) [53]; mouse monoclonal anti-Oct3/4 (1:300, Santa Cruz, sc-5279); goat polyclonal anti-Lamin B (1:200, Santa Cruz, sc6216). Alexa fluor-conjugated (Alexa488, Alexa555, Alexa647) secondary antibodies (Molecular Probes, Thermo Fisher Scientific, Waltham, MA) were incubated for 1 h at RT in 5% BSA, the blastocysts were washed twice, incubated for 30 min with 0.3 μM DAPI in PBS, washed again, and finally stored at 4 °C in PBS in a glass bottom culture dish (MatTek, Ashland, MA) until imaged.

### Indirect immunofluorescence staining of ES cells in vitro

Following treatment with RA, cells were plated onto glass coverslips and incubated for 2 more days in the presence of 1 μM RA. For immunofluorescence, cells were fixed in 4% paraformaldehyde in PBS for 15 min at RT, washed 1X with PBS, followed by permeabilization with 0.1% Triton X-100 for 5 min at RT. Fixed cells were blocked for 1 h at RT with 5% BSA (Fraction V; Calbiochem/EMD Millipore, Billerica, MA) in PBS. Primary antibodies were diluted in 5% BSA: mouse monoclonal anti-Lamin B (1:300, Santa Cruz, sc373918; Dallas, TX); rabbit polyclonal anti-Gata4 (1:400, Santa Cruz, sc9053); goat polyclonal anti-Lamin B (1:400, Santa Cruz, sc6216); mouse monoclonal anti-Oct3/4 (1:400, Santa Cruz, sc5279); rabbit anti-Nanog (1:400, Abcam, ab80892, Cambridge, MA); rabbit polyclonal anti-Gata6 (1:1000, [49]). The anti-Lamin B antibody recognizes Lamin B1 primarily and to a lesser extent Lamin B2, of mouse, rat, and human origin, according to the manufacturer’s data sheet. After incubating overnight at 4 °C, coverslips were washed 5 times for 5 min each with PBS containing 0.05% Tween-20, and secondary antibodies were added for 1 h at RT. Secondary antibodies used were Alexa fluor (488 or 555)-conjugated donkey anti-mouse, anti-rabbit, or anti-goat antibodies from Molecular Probes (Thermo Fisher Scientific). The coverslips were washed extensively, counterstained for 2 min with 0.3 μM DAPI, and mounted in ProLong Gold (Thermo Fisher Scientific).

### Confocal microscopy and analysis

Laser scanning confocal microscopy was performed on a Zeiss Imager.M2 LSM 700 using the plan-Apochromat 63X/1.4 NA oil DIC M27 objective, and imaged using the Zeiss Zen software. Each individual two-dimensional sectional image in the *z*-stack was 512 × 512 pixels, the scaling was set at 0.4 μm, and the step size was 0.38 μm/pixel [60]. The *x*- and *y*-coordinates were determined using the Lamin B and/or DAPI images and when appropriate the Gata4 image at the largest diameters that were perpendicular with respect to the other, for individual nuclei using the AxioVision measurement tool. *Z*-coordinates were determined from the top to the bottom of the same nuclei. The number of *z-*steps was multiplied by the step size (0.38 μm) to calculate the depth of the nucleus. All volume measurements used the formula approximating an ellipsoid shape: $\boldsymbol{V} = \mathbf{\mathsf{4}}/\mathbf{\mathsf{3}}\ \boldsymbol{\pi} \boldsymbol{abc} = \boldsymbol{\pi} /\mathbf{6}\ \boldsymbol{x}\boldsymbol{y}\boldsymbol{z}$, where *a*, *b*, and *c* are the radii of the *x*, *y*, and *z* dimensions, respectively [38]. The models were created using SolidWorks 2010. Student’s unpaired t-test calculator offered online from GraphPad was used to test for significance, calculated as the two-tailed *P*-value, where *P* < 0.05 is considered statistically significant. Results are plotted in the graphs as mean ± standard deviation (s.d.). For ES cell measurements, we also used Volocity® 3D Image Analysis Software from Perkin Elmer (Thermo Fisher Scientific), to quantitate volume, surface area, and shape contour factor based on the nuclear outline provided by Lamin B or Gata4, where present. Shape factor represents the degree of roundness and smoothness of a structure and is defined as: ***Shape Factor*** = **4** ***π*** (***nuclear area***)/(***nuclear perimeter***)^**2**^. Shape factor approaches 1 for a perfectly smooth spherical nucleus and goes towards 0 for an elongated or convoluted nucleus [37, 61].

### Immunoblot analysis

ES cells were treated with 1 μM RA for 4 days. The cells were lysed in RIPA buffer containing protease and phosphatase inhibitors, and protein concentration was measured by BioRad-DCC protein assay. The lysates were diluted 1:1 with BioRad 2X loading buffer, and equal protein was loaded on 4-12% Tris glycine-polyacrylamide (Invitrogen) gels. Separated protein was electrotransferred to nitrocellulose membrane (Pall, Pensacola, FL), and immunoblotted with primary antibodies. Antibodies used were: mouse monoclonal anti-lamin A/C (1:1000, Active Motif, #39288); rabbit polyclonal anti-emerin (1:1000, ProteinTech, #10351-1-AP); mouse monoclonal anti-actin (1:5000, BD-Transduction, #612656); rabbit polyclonal anti-ERK2/1 (1:1000, Cell Signaling, #9102); mouse monoclonal anti-Dab2 (1:2000, BD-Transduction, #610464); rabbit polyclonal anti-Gata4 (1:1000, Santa Cruz, sc9053); mouse monoclonal anti-Oct3/4 (1:1000, Santa Cruz, sc5279); rabbit anti-Nanog (1:1000, Abcam, ab80892); mouse monoclonal anti-N-cadherin (1:1000, BD-Transduction, #610920). HRP-conjugated secondary antibodies were from BioRad and used at 1:5000 dilution. SuperSignal West Dura Extended Duration substrate (Thermo Scientific, #34076) was used to visualize the immunoblots.

### RT-PCR expression analysis

Wild type and mutant ES cells were cultured on 10-cm gelatinized tissue culture dishes in the absence (minus RA) or presence of 1 μM RA (plus RA) for 4 days, and total RNA was harvested from ES cells using the Qiagen RNAeasy kit. cDNAs were prepared from the extracted RNA by reverse transcription with iScript™ cDNA Synthesis Kit according to the manufacturer’s instructions (BioRad, Hercules, CA). Quantitative RT-PCR was performed using BioRad CFX Connect™ Real-Time PCR Detection System with 2X SYBR® Green Supermix and specific primers. The primers used were purchased from Integrated DNA Technologies (Coralville, IA): *GATA4f*: 5' GCC TCT ATC ACA AGA ACG GC; *GATA4r:* 5’ TAC AGG CTC ACC CTC GGC ATT A; *GATA6f*: 5’ ATC CGG TCT CTA CAG CAA GAT GA; *GATA6r*: 5’ CGC CAT AAG GTA GTG GTT GTG G; *Cdx2f:* 5’ CAT CAG GAG GAA AAG TGA GCT GG; *Cdx2r*: 5’ TTT TCC TCT CCT TGG CTC TGC A; *Cdh5f*: 5’ TGG TCT TGC GGA TGG AGT A; *Cdh5r*: 5’ CAG CGA CAC TTC TAC CAC TTC. GAPDH and Lamin B1 were IDT PrimeTime predesigned primers Mm.PT.39a.1 and Mm.PT.56a, respectively. RT-PCR results were analyzed using the BioRad CFX software. Results for each sample have been normalized to respective GAPDH expression, and plotted relative to control wild type (WT). For Gata6, Gata4, Cdh5, Cdx2, and Lmnb1, expression is plotted relative to untreated WT (minus RA). Oct3/4 and Nanog are pluripotency markers; Gata6 and Gata4 are endoderm markers; Cdx2 (caudal-type homeobox transcription factor 2) marks extra-embryonic ectoderm and trophectoderm lineages; and Cdh5 (VE-cadherin, or vascular endoderm cadherin) marks the endothelial lineage [62].

### Transmission electron microscopy

Embryoid bodies formed from the different ES cells were harvested after 5 days of treatment with 1 μM retinoic acid. After collection, they were immediately fixed and processed for transmission electron microscopy by the University of Miami Miller School of Medicine electron microscope according to standard protocol, as described in detail previously [58].

## Additional files

Additional file 1: Figure S1. Confirmation of reduced primitive endoderm differentiation of ES cells deficient of lamin A/C and/or emerin. ES cells were incubated with 1 μM RA for 4 days, and cell lysates were prepared. Two different groups of cell lysates were analyzed. These lysates were prepared at different times during the course of the study, approximately 7 months apart. Protein loading was normalized to total (pan) ERK1/2. To validate the antibody used in the experiments shown in Fig. 3, two additional, different antibodies to Lamin A were used to detect lamin A specifically [Abcam rabbit polyclonal antibody #26300] or lamin A/C [Cell Signaling Technology (CST) #2032]. **a** A Western blot shows the absence of lamin A/C and/or emerin proteins in ES cell lines with *lmna* (−/−) and/or *emd* (−/−) genotypes. It should be noted that the anti-Lamin A/C antibody (shown in the top panel) generates a non-specific band that migrates between Lamin A and Lamin C. This band is found in WT and all mutant samples. **b** To observe any weak signal, the immunoblots in “a” were exposed longer to the film before developing. In Group A, the *emd −/−* cells have only weak Lamin A/C expression; in Group B, Lamin A/C is virtually undetectable. (PPTX 558 kb)
Additional file 2: Figure S2. Indirect immunofluorescence detection of lamin A and emerin in ES cells. ES cells were incubated with 1 μM RA for 4 days to induce endoderm differentiation. Cells were fixed and processed for immunofluorescence as described in “Methods.” Primary antibodies used were rabbit polyclonal anti-lamin A (Abcam #26300) and goat polyclonal anti-emerin (Santa Cruz #sc-8086). Secondary antibodies used were Alexa-555-conjugated donkey anti-rabbit and Alexa-488-conjugated donkey anti-mouse antibodies (Molecular Probes, Thermo Fisher Scientific). Nuclei were counterstained with DAPI. Merged images are shown. In the WT sample, for clarity, the box inset shows only immunofluorescence staining for emerin. These images confirm the absence of expression of lamin A and emerin in the mutant ES cells and the low expression of lamin A/C in the *emd −/−* cells. (PPTX 487 kb)

## Abbreviations

E4.5: Embryonic day 4.5
ES: Embryonic stem
INM: Inner nuclear membrane
ONM: Outer nuclear membrane
RA: All-trans retinoic acid
wt: Wild type
