# Supplementary figures and images for: Nuclear envelope structural proteins facilitate nuclear shape changes accompanying embryonic differentiation and fidelity of gene expression
Source: BMC Cell Biol. 2017 Jan 14;18:8. doi: 10.1186/s12860-017-0125-0 (PMC5237523; doi:10.1186/s12860-017-0125-0)

## Slide 1
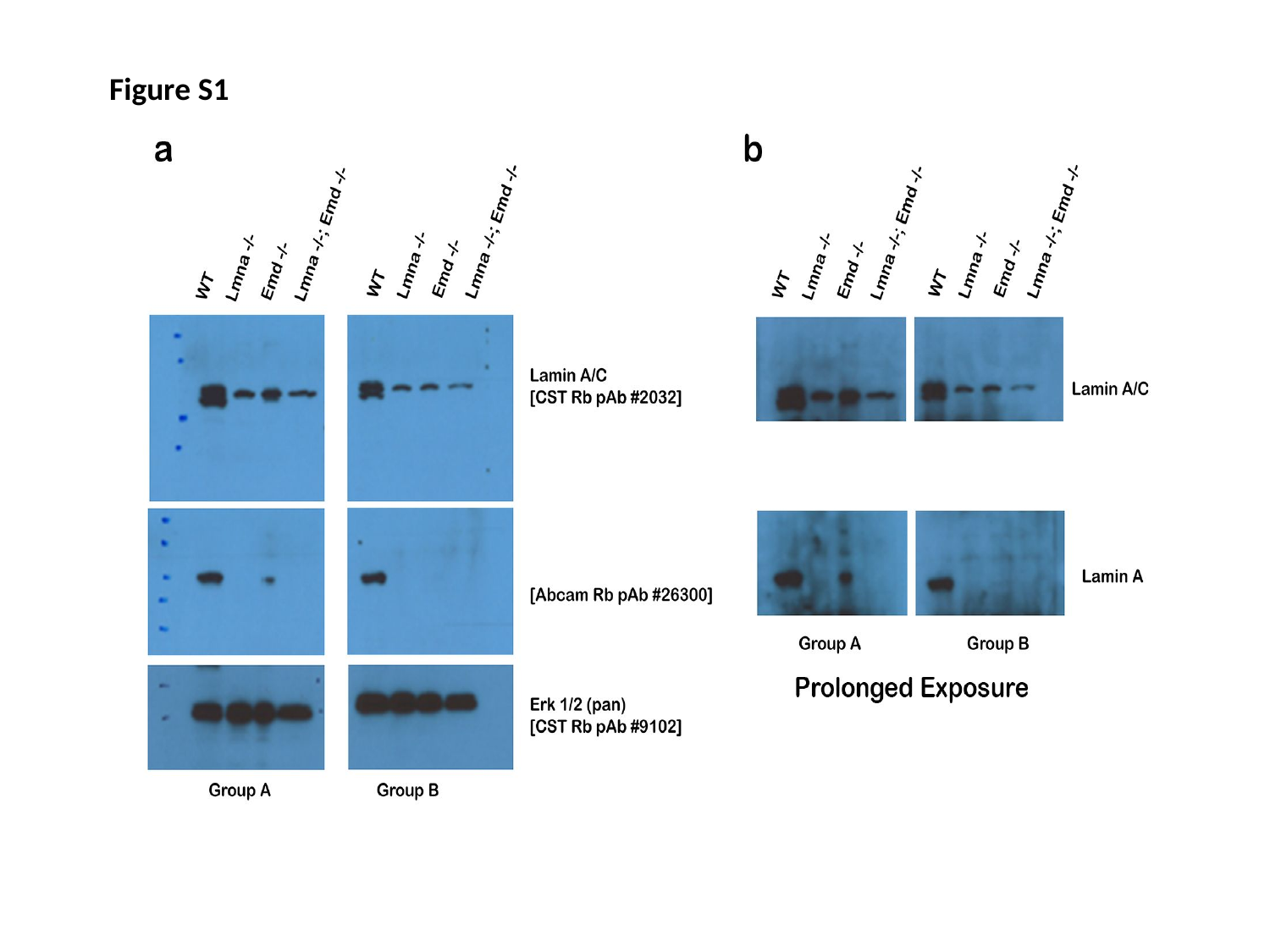

Figure S1

Supplement: Additional file 1: Figure S1. — Confirmation of reduced primitive endoderm differentiation of ES cells deficient of lamin A/C and/or emerin. ES cells were incubated with 1 μM RA for 4 days, and cell lysates were prepared. Two different groups of cell lysates were analyzed. These lysates were prepared at different times during the course of the study, approximately 7 months apart. Protein loading was normalized to total (pan) ERK1/2. To validate the antibody used in the experiments shown in Fig. 3, two additional, different antibodies to Lamin A were used to detect lamin A specifically [Abcam rabbit polyclonal antibody #26300] or lamin A/C [Cell Signaling Technology (CST) #2032]. a A Western blot shows the absence of lamin A/C and/or emerin proteins in ES cell lines with lmna (−/−) and/or emd (−/−) genotypes. It should be noted that the anti-Lamin A/C antibody (shown in the top panel) generates a non-specific band that migrates between Lamin A and Lamin C. This band is found in WT and all mutant samples. b To observe any weak signal, the immunoblots in “a” were exposed longer to the film before developing. In Group A, the emd −/− cells have only weak Lamin A/C expression; in Group B, Lamin A/C is virtually undetectable. (PPTX 558 kb) [file 12860_2017_125_MOESM1_ESM.pptx]

## Slide 1
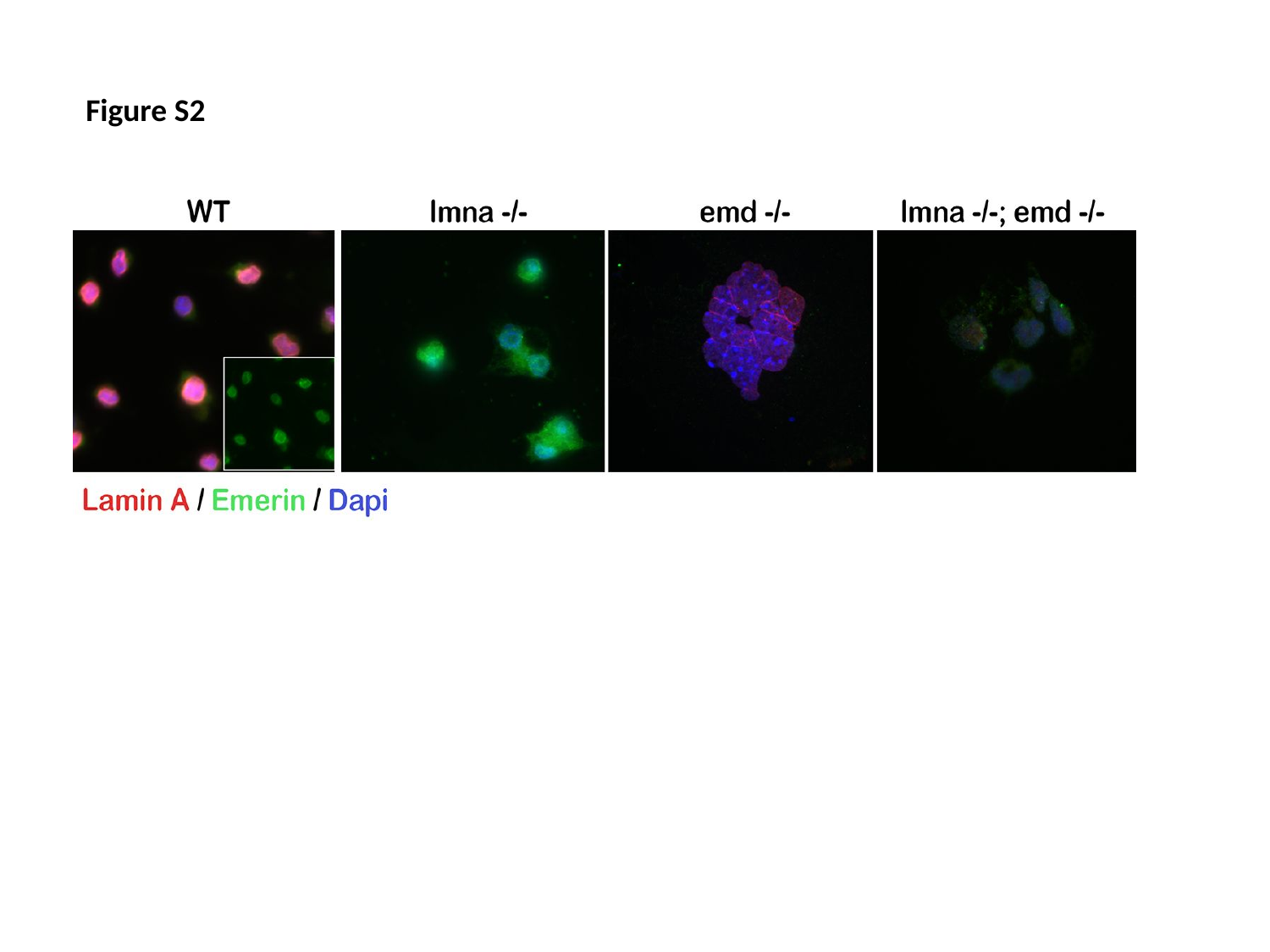

Figure S2

Supplement: Additional file 2: Figure S2. — Indirect immunofluorescence detection of lamin A and emerin in ES cells. ES cells were incubated with 1 μM RA for 4 days to induce endoderm differentiation. Cells were fixed and processed for immunofluorescence as described in “Methods.” Primary antibodies used were rabbit polyclonal anti-lamin A (Abcam #26300) and goat polyclonal anti-emerin (Santa Cruz #sc-8086). Secondary antibodies used were Alexa-555-conjugated donkey anti-rabbit and Alexa-488-conjugated donkey anti-mouse antibodies (Molecular Probes, Thermo Fisher Scientific). Nuclei were counterstained with DAPI. Merged images are shown. In the WT sample, for clarity, the box inset shows only immunofluorescence staining for emerin. These images confirm the absence of expression of lamin A and emerin in the mutant ES cells and the low expression of lamin A/C in the emd −/− cells. (PPTX 487 kb) [file 12860_2017_125_MOESM2_ESM.pptx]
